# HIV cure strategies: which ones are appropriate for Africa?

**DOI:** 10.1007/s00018-022-04421-z

**Published:** 2022-07-06

**Authors:** Christopher Zaab-Yen Abana, Helena Lamptey, Evelyn Y. Bonney, George B. Kyei

**Affiliations:** 1 Department of Virology, College of Health Sciences, Noguchi Memorial Institute for Medical Research, University of Ghana, Accra, Ghana; 2 Department of Immunology, College of Health Sciences, Noguchi Memorial Institute for Medical Research, University of Ghana, Accra, Ghana; 3 Departments of Medicine and Molecular Microbiology, Washington University in St. Louis, 660 S. Euclid Ave, St. Louis, MO USA; 4 Medical and Scientific Research Center, University of Ghana Medical Centre, Accra, Ghana

**Keywords:** HIV cure, HIV latency, Reservoir, Africa

## Abstract

Although combination antiretroviral therapy (ART) has reduced mortality and improved lifespan for people living with HIV, it does not provide a cure. Patients must be on ART for the rest of their lives and contend with side effects, unsustainable costs, and the development of drug resistance. A cure for HIV is, therefore, warranted to avoid the limitations of the current therapy and restore full health. However, this cure is difficult to find due to the persistence of latently infected HIV cellular reservoirs during suppressive ART. Approaches to HIV cure being investigated include boosting the host immune system, genetic approaches to disable co-receptors and the viral genome, purging cells harboring latent HIV with latency-reversing latency agents (LRAs) (shock and kill), intensifying ART as a cure, preventing replication of latent proviruses (block and lock) and boosting T cell turnover to reduce HIV-1 reservoirs (rinse and replace). Since most people living with HIV are in Africa, methods being developed for a cure must be amenable to clinical trials and deployment on the continent. This review discusses the current approaches to HIV cure and comments on their appropriateness for Africa.

## Introduction

**Fig. 1:**
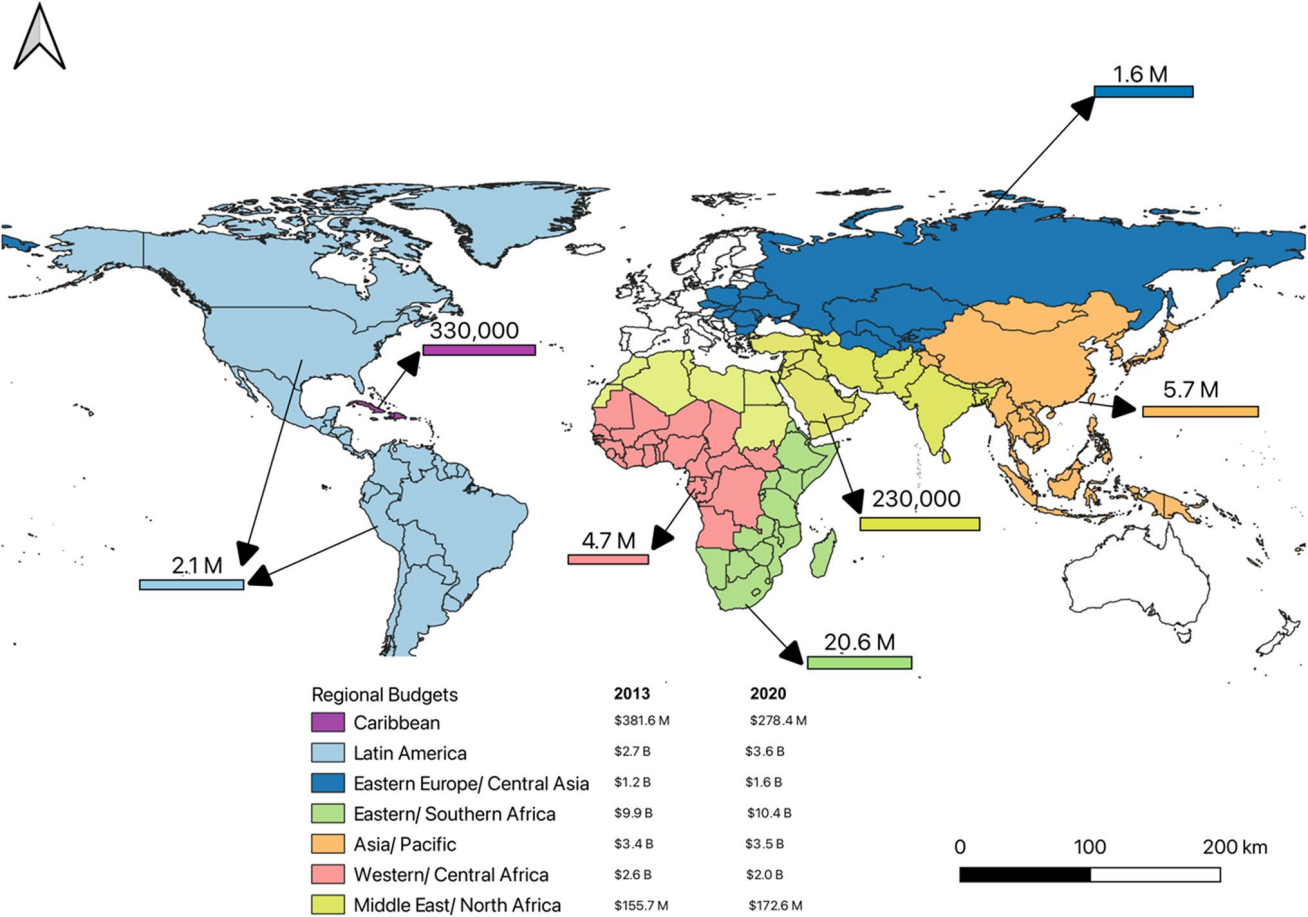
A world map indicating the regional HIV infections and breakdown of resources available for treating HIV at two-time points (2013 and 2020). Source of data: UNAIDS Financial Dashboard, 2021

The HIV pandemic remains one of medicine’s greatest challenges with an estimated 38.0 million people living with HIV (PLWH) of which the vast majority (25.7 million) are in Africa [1]. While antiretroviral therapy (ART) can halt viral replication, reduce mortality, and improve the lifespan of PLWH, this treatment is lifelong, expensive, inaccessible to many, and cannot eradicate the latent virus [2, 3]. It is difficult to find a cure for HIV due to the persistence of latently infected cells that produce the virus following interruption of ART [4, 5], presence of long-lived HIV-infected resting memory CD4 + T cells [6–10], the exhaustion of HIV-specific CD8 + T cells [11, 12] and the difficulty in reaching anatomic sanctuary sites by HIV-specific CD8 + T cells [5, 13]. More so, the current World Health Organization (WHO) eligibility criteria of treating all regardless of CD4 count has increased the funding gap for ART [14]. In addition, with the decline of global funding for HIV [15] and the negative impact of the COVID-19 pandemic on the world economy, sustaining existing HIV/AIDS treatment programs is becoming even more challenging making the quest for a cure more acute. Between 2013 and 2020, despite considerable increase in the number of patients on ART, HIV funding has been generally flat or reduced (Fig. 1).

Three categories of HIV cure approaches have been identified: eradication cure (elimination of all viral reservoirs), functional cure (immune control without reservoir eradication), or a hybrid cure (reservoir reduction with improved immune control) [16]. Approaches to HIV cure under investigation include boosting the host immune system, genetic approaches to disable co-receptors or the viral genome, modification of host cells to resist HIV, and engineered T cells to eliminate HIV-infected cells. Others include therapeutic vaccination, broadly neutralizing antibodies, purging cells harboring latent HIV with latency-reversing agents (LRAs), preventing replication of latent proviruses (block and lock) and boosting T cell change to reduce HIV-1 reservoirs (rinse and replace) [2, 3, 17–24]. As HIV cure research advances, it is important to bear in mind that the vast majority of PLWH who will need these cures live in Africa where resources are limited. Therefore, there is a need to design cure strategies that will be feasible for clinical trial and implementation in Africa. This article reviews ongoing HIV cure approaches, discusses their pros and cons, and suggests how they could be made suitable for Africa.

## Progress towards HIV cure

Tremendous successes have been chalked in the fight against HIV since its identification forty years ago [25]. Globally, ART is now available to about 70% of PLWH, thus increasing life span and reducing deaths due to HIV/AIDS [1]. The hope to find a cure for HIV was high after initial reports of both eradication and functional cures. Timothy Brown, the Berlin patient, was the first reported case of eradication cure. He received a bone marrow transplant for acute myeloid leukemia from a donor who was negative for HIV and had a mutation in the CCR5 co-receptor, which is required for HIV entry. He lived free of HIV for close to 10 years [26] and died in 2020 from complications of leukemia. After unsuccessful attempts to replicate the same procedure [27, 28], there was a success in another individual, the London patient, who achieved long-term suppression of HIV-1 [29].

The first reported case of a functional cure was the Mississippi baby who was born to an HIV-positive mother and given a full dose of ART 30 h after birth. Although the family interrupted ART for 18 months, surprisingly HIV remained undetected in the blood [30]. Notwithstanding, there was a rebound of detectable HIV in plasma after 27.6 months [31]. Subsequently, the VISCONTI (Virological and Immunological Studies in Controllers after Treatment Interruption) study in 2013 implied that early initiation of ART enables some patients to maintain a low viral load after ART interruption [32]. Since these initial reports, there has been growing interest in developing curative therapies for HIV.

## HIV cure strategies

### Stem cell transplants

Ferrebee and Thomas [33] pioneered stem cell therapy which has now become a platform for treating leukemias, lymphomas, and many other malignancies. In people living with HIV, the procedure was first carried out in the 1980s oblivious of the virus since there were no assays to detect HIV [34]. Following this report, several attempts were made to treat HIV using this approach but they were not successful [35–38]. However, the Berlin patient gave a glimpse of hope that achieving a virus-free state in the absence of ART is possible. Despite that, stem cell transplant for treating HIV is not practical for several reasons. First, stem cell transplant is a high-risk procedure with a high mortality rate and thus only employed in the most desperate situations such as uncontrolled leukemia. The conditioning and bone marrow ablation required for the procedure put the patient at great risk of dying from infections. Second, even in developed countries, the procedure is performed in the most specialized centers where specially trained oncologists, geneticists, hemato-pathologists, and infectious disease specialists are available. These are resources that are mostly not available in Africa. Third, the relative paucity of potential CCR5∆32 donors (less than 1% of Caucasians) make it unattractive as a potential therapy. Thus, while stem cell transplants indicate that HIV is potentially curable, it is not a practical strategy for ending the AIDS pandemic.

### Shock and kill

**Fig. 2:**
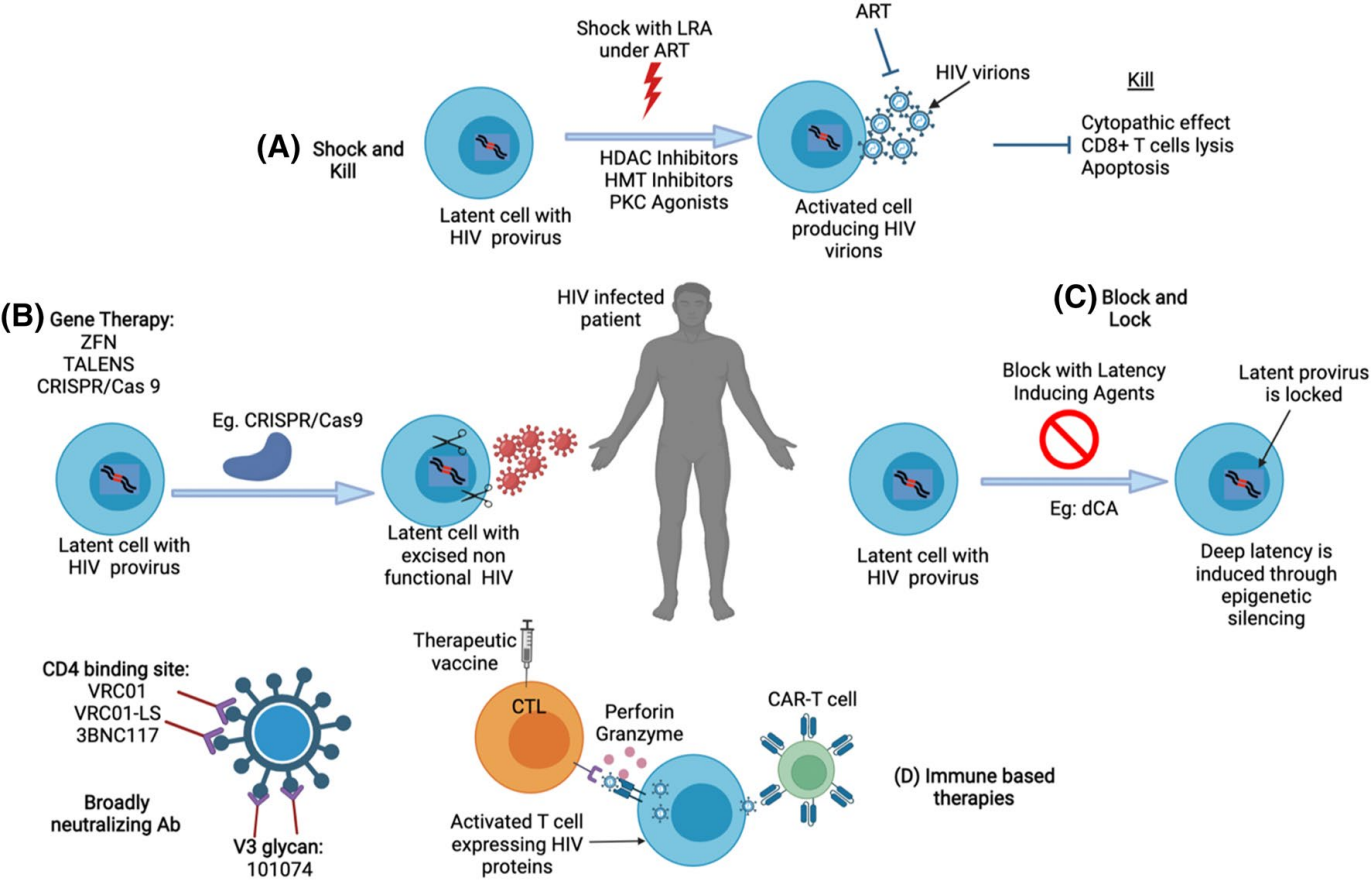
Strategies under development for an HIV cure. **A** Shock and kill approach using latency-reversing agents (LRAs) to eradicate the latent reservoir. **B** Gene therapy utilizing CRISPR to target the latent reservoir. **C** Block and lock approach using latency-inducing agents to induce silencing of the latent reservoir. **D** Immune-based therapies using therapeutic vaccines, CAR-T cells, and broadly neutralizing antibodies. *HDAC* Histone deacetylase, *HMT* histone methyl transferase, *PKC* protein kinase C, *dCA* didehydro-cortistatin A, *ART* antiretroviral therapy

The shock and kill approach uses compounds known as latency-reversing agents (LRAs) to reactivate the latent provirus. The premise is that patients will be given agents that reactivate the latent virus in the resting CD4 + T cells whiles they are on ART (Fig. 2). Since viral replication is usually toxic to CD4 + T cells, it is expected that reactivation will result in the death of these cells, and because patients will be on ART at this stage, any virus produced will not be able to infect bystander cells. In addition, once viral production begins in the resting T cells, they will be recognized by the immune system for clearance. With the clearance of the infected resting T cells, patients could stop taking ART and undergo occasional monitoring. The concept of the shock and kill approach is hinged on reducing the size of the latent reservoir and limiting viral rebound [39]. In theory, any agent that can stimulate resting T cells enough for viral production could be an LRA. Interleukin 2 (IL-2), anti-CD3 antibody, and TNF*α* were the first LRAs tested in HIV-infected individuals receiving ART [40, 41], however, the results were not promising. The T cell receptor agonist and PMA (phorbol 12-myristate 13-acetate) were used but resulted in global T cell activation. Therefore, an ideal LRA should be able to cause proviral latency reversal without global T cell activation. Latency-reversing agents (LRAs) have been categorized based on their mechanism of action as shown in Table 1 [39]. Of the six categories, the histone deacetylase inhibitors (HDACis) have received much attention with several small early clinical trials completed [39, 42–45]. Although the shock and kill approach is the most clinically advanced cure strategy, clinical studies have focused mainly on HDACis which are already approved for cancer therapy. In these trials, some adverse effects have been reported [46]. These include but not limited to nausea, vomiting, anorexia [47–50]; fatigue [51–53]; and skin changes [54]. HDACis have been the focus of many researchers and investigated extensively as a potential LRA [55–57]. However, several groups have shown that one LRA might not be effective in reducing the size of the reservoir either due to an inadequate reactivation or lack of an effective kill or both [39, 58, 59] and suggested a combination of two or more LRAs to achieve robust viral reactivation and a significant reduction in reservoir size [60–65]. Concerning potential combination LRA treatment, one of the most favored is a combination of HDACi and PKC agonists like bryostatin [66, 67]. However, there are fears of severe side effects with bryostatin. Therefore, the idea is to use a lower concentration of bryostatin to synergize with HDACis to achieve potent reactivation while reducing the chances of adverse events. That said, this combination is yet to be tested in clinical trials. Even though, the combination increases the reactivation potential, several obstacles exist in the killing of the reactivated cells. This includes the resistance of the cells to apoptosis, exhaustion of CD8 + cells, and immune escape mutation in chronically infected individuals [68]. Nonetheless, Herzig and his colleagues recently proposed a more effective kill of reactivated cells by utilizing chimeric antigen receptors (CAR) coupled with broadly neutralizing antibodies in an ex vivo study [69].

**Table 1:** Examples of latency-reversing agents (LRAs) categorized into six different primary classes according to their mechanisms of action

Class	Mechanism of action	Types	Examples	References
Histone post-translational modification modulators	They modify the histone tails in the nucleosomes of the integrated HIV genome thus making them susceptible to reactivation. The HDAC inhibitors and histone methyltransferase (HMT) are a major components of this category	HMT (SMYD2)	A2391	[70]
		KAT5 inhibitor	MG-149	[71]
		HDAC (Class 1) inhibitor	Romidepsin, Entinostat (MS-275), Largazoles (SDL148; JMF1080; SDL256)	[72–74]
		HDAC (Pan) inhibitor	Vorinostat, Givinostat (ITF2357), Belinostat (PXD101), Panobinostat (LBH589), Valproic Acid (VPA)	[55, 75–77]
		Polycomb (L3MBTL1) inhibitor	UNC-926	[70]
Non-histone chromatin modulators	They modulate other elements present in the chromatin, for, instance other transcription factors other than NF-κB, DNA methylation, and various functional proteins	BAF inhibitors	CAPE; MGD-486; Pyrimethamine	[78–80]
		Brd4 inhibitors	8-Methoxy-6-methyl quinoline-4-ol (MMQO), JQ1	[81, 82]
		DNMT inhibitors	Decitabine (5-aza-2′-deoxycytidine), Zebularine	[83, 84]
		NFAT pathway activator	AV6	[85]
		STAT5 sumoylation inhibitors	Benzotriazoles (HODHBt, HBt, HOBt, HOAt)	[86]
NF-kB stimulators	The most robust LRA. The major component of this group is the protein kinase C (PKC) pathway agonists. The pathway leads to the activation of NF-κB activation thus resulting in HIV transcription and reactivation	SIRT2 inhibitor	AGK2	[87]
		SMAC mimetics	CAPE; MGD-486; Pyrimethamine	[78]
		PKC Agonists	Bryologs, Bryostatin-1, Phorbol 12-myristate 13-acetate (PMA), Prostratin, Ingenol-B (ingenol-3-hexanoate)	[88–93]
Toll-like receptor agonists	Stimulate TLR-based innate immune system through different pathways to cause transcription of the HIV provirus	TLR2 agonists	HKLM	[94]
		TLR2/7 agonists	CL413	[95]
		TLR5 agonist	Flagellin	[64]
		TLR7/8 agonist	R-848	[96]
		TLR8 agonist	3 M-002	[97]
		TLR9 agonists	CPG-7909	[98]
Extracellular stimulators binding	They operate by exerting their effect via their receptors extracellularly. It includes all compounds and molecules	CCR5	Maraviroc	[99, 100]
		CD28	αCD28	[101]
		TCR agonist	αCD3	[101]
		TNF Receptor agonist	TNFα	[89]
		Surface glycoproteins	Phytohemagglutinin (PHA)	[102]
Miscellaneous	Have an unknown/unconfirmed mechanism of action. Compounds that are not common and function by modulating unique cellular mechanisms	BTK inhibitor	Terreic acid	[103]
		Calcineurin agonist	Ionomycin	[104]
		PI3K agonist	Oxoglaucine (57,704)	[105]
		PKA agonist	Bucladesine (dibutyryl-cAMP)	[106]
		Unknown	Piceatannol, Quinolin-8-ol derivatives, HHODC	[103, 107, 108]

Another scenario where the T cells could be stimulated is the “Rinse and Replace” approach [24]. This is a yet to be tested approach which proposes that under ART, T cell activation could be induced to produce waves of polyclonal T cell differentiation resulting in a situation where latently infected cells are replaced by new uninfected cells. The infected cells are continually “washed out” due to the continuous activation, differentiation, and cell death. This approach is different from the shock and kill approach in that it promotes physiological replacement of frequently activated cells. In addition, only a proportion of latently infected cells are required to express virus that provides adjuvant effects thus helping to induce a potent flux. Furthermore, this functional cure approach aims to combine other cure approaches such as block and lock to prevent viral rebound in the absence of ART. The approach also strongly recommends several rounds of ART and starting ART on time thus raising the concern of negative side effects, adherence, access to ART and cost. Implementing this approach in Africa will be problematic because of the concerns raised on ART. Moreover, clinical trials regarding this approach are yet to commence.

The shock and kill approach could be easily deployed in resource limited settings like Africa, since all that is required is adding another agent to the current ART regimen. That said, an oral regimen will be more preferable to intravenous infusions in an African setting.

The figure was Created with BioRender.com.

### Block and lock

The block and lock approach proposes the use of small interfering RNAs (siRNA) [20, 112, 113] or latency-inducing agents [114, 115] to effect transcriptional silencing (block) at the HIV promoter using epigenetic mechanisms to lock the integrated viral genome in a permanent position thereby preventing transcription of new virions even when ART is stopped (Fig. 2). The HIV transactivator of transcription (Tat) protein has been an important target for this approach since it is essential for transcription initiation and elongation. The tat protein which is produced early during the life cycle of HIV promotes the transcription of HIV by binding to the transactivation response element (TAR) and recruiting the positive transcription elongation factor B (P-TEFb) to promote transcriptional elongation [115]. By far the most advanced strategy of the block and lock has been the use of a Tat inhibitor didehydro-cortistatin A (dCA) to enforce HIV latency [21, 115–119]. This compound has shown remarkable success in maintaining viral latency ex vivo in primary T cells from virologically suppressed patients and in vivo in mouse models. In both instances, dCA delayed viral rebound when ART was interrupted [21, 120, 121]. Similar results for dCA were reported in HIV-2 and Simian Immunodeficiency Virus (SIV) [122]. With no obvious adverse effects and the ability to cross the blood brain barrier, dCA may be able to reach the brain and other sanctuary sites of the body where HIV resides [123]. However, induction of resistance to dCA in the laboratory has been reported [124] making it worthwhile to investigate if some strains of HIV may be resistant to the compound. Although there is evidence that dCA induces epigenetic modification of the HIV promoter, the mechanism is not well understood.

Triptolide (TPL), which is predominantly used for the treatment of rheumatoid arthritis, inhibits the function of Tat, thereby promoting latency [125]. Others found that TPL blocks RNAPII and prevents it from initiating transcription [126]. However, the ability of this molecule to interfere with important cellular functions limits its clinical use [118]. Another inhibitor of Tat-mediated viral transcription is Levosimendan, an FDA-approved drug for the treatment of heart-related conditions [127]. The effects of other inhibitors targeting host factors or signaling pathways required for the transcription of viruses, such as P-TEFb, heat shock protein 90 (HSP90), mammalian target of rapamycin (mTOR) complex, facilitates chromatin transactions (FACT), bromodomain-containing protein 4 (BRD4), and xeroderma pigmentosum subtype B (XPB) is reviewed elsewhere [118, 120, 128]. The absence of cellular homologs and the limited off-target effects when Tat-TAR is inhibited makes it an outstanding target for the block and lock approach [119]. The block and lock strategy targets long-term remission of HIV in a more specific manner without global T cell activation, compared to some non-specific shock and kill strategies. Even though the block and lock approach is potentially scalable and could be deployed in Africa if successful, it still awaits human trials to determine feasibility.

### Gene therapy to eradicate HIV reservoirs

Innovative ways targeting the genome of HIV in a bid to find a cure for the infection have advanced over the past decades. The idea of gene editing therapy is to alter a selected gene locus to change or interrupt its normal function using engineered nucleases (Fig. 2). This results in deletions or additions at the selected gene target site [22, 129]. Two main repair pathways are involved: (i) non-homologous end-joining (NHEJ) where the break ends are directly ligated without a homologous template and (ii) homology-directed repair pathway (HDR) in which homologous sequences are introduced to guide the repair [130]. The HDR yields limited off-target genome effects [131]. Forms of potential gene editing-based HIV therapies include zinc finger nuclease (ZFN), transcription activator-like nucleases (TALENS), and clustered regularly interspaced short palindromic repeats (CRISPR)-associated protein 9 (CRISPR/Cas9).

The most tried gene therapy approach targeting HIV infection is the ZFN [132]. It is made up of two domains; the *FokI* endonuclease for cleaving target sequences and the Cys2-His2 zinc-finger proteins (ZFPs) for specific DNA-binding [133, 134]. The initial report utilizing ZFN targeting the C–X–C chemokine receptor type 4 gene (CXCR4) disruption displayed promising results [135, 136]. Given that the mutant CCR5∆32 protein confers resistance to HIV infection and the Berlin patient was cured using a ∆32 stem cell transplant, the CCR5 became an ideal target for HIV gene therapy. Following the initial use of the ZFN approach to disrupt the CCR5 in HIV-infected cells [137, 138], several other works have built upon this strategy [138–141]. More so, there is evidence that ZFN could be used to disrupt the CCR5 in human induced pluripotent stem cells and human embryonic stem cells [142]. Similarly, HIV resistance was achieved when mice were treated with CCR5-disrupted gene hematopoietic stem cells [139]. In a landmark safety study in humans, Tebas et al. used ZFN to edit CCR5 out of CD4 + T cells isolated from HIV patients, and reinfused into the same patients showing that the procedure was safe [143].

In contrast to ZFN, TALENs are engineered proteins that can cleave dsDNA sequences in a single base pair modular fashion [144, 145]. This presupposes that they can bind to wider DNA targets than ZFN. The DNA binding proteins are derived naturally from Xanthomonas, a plant bacterial pathogen [146]. Clinically, TALENS are yet to be applied for the treatment of HIV. However, the success of several experimental studies indicates that it can be scaled up for HIV [147–149]. In comparison to ZFNs, TALENs are cost-effective but difficult to generate, bulkier and the delivery to several cell targets is challenging [150]

The CRISPR/Cas9 uses short-guide RNA (gRNA) to target a specific DNA sequence after cleaving the double-stranded DNA by the Cas9 endonuclease. The double-stranded DNA is then repaired by NHEJ or homologous recombination [151]. The CRISPR/Cas9 approach is being widely investigated as a tool to combat various diseases [152–155]. Ebina et al. first applied this technology to HIV in cell culture to target the integrated provirus [156]. Subsequently, several scientists have explored the potential of the CRISPR/Cas9 technology to target the virus in cell culture systems and mouse models [157–162]. The versatility of the CRISPR/Cas9 technology is such that it can also be used to target co-receptors, restriction factors, and proteins that promote or inhibit HIV latency. It has also been demonstrated that the CCR5 could be silenced in a human embryonic kidney (HEK) 293 T cells transfected with Cas9 and sgRNAs [163]. Subsequently, the piggyBac technology was used to enhance a homozygous ∆32 mutation in induced pluripotent stem cells via the CRISPR/Cas9 system [158]. Wang and his research group also knocked out CCR5 co-receptors using lentivirus vectors to express CCR5-sgRNA and Cas9 [157]. The CXCR4 co-receptor has also been disrupted by the CRISPR/Cas9 technology in CD4 + T cells of humans and rhesus macaque [159]. In addition, this technology has been explored to reactivate HIV. Scientists have used deficient Cas9 (dCas9) coupled with transcription activator domains to trigger the transcription of HIV in latent reservoir cells [164–167].

Since host restriction factors are weakly expressed during HIV infection [168, 169], CRISPR/Cas9 has been utilized in the activation of the expression of these enzymes. For instance, the technology was used in cell culture systems to induce the expression of APOBEC3G (A3G) and APOBFC3B (A3B) to inhibit HIV infection [170]. Disadvantages of the CRISPR/Cas9 technology include potential off-target effects that could induce gene mutations, and the lack of an effective delivery system [164, 166, 169, 171–173]. While adenoviral vectors have been effective in delivering CRISPR/Cas9 [174, 175], lentiviral mode of delivery could increase the risk of off-target effects [157, 176]. Cytotoxicity and immune tolerance are also limitations in the use of the CRISPR-based technology in the fight against HIV [173].

If successful, could gene therapy be widely deployed in Africa? The answer will depend on several key factors. Methods like taking CD4 + T cells from patients, modifying them in the laboratory, and reinfusing them into the same patient (autologous transplant) will be challenging to implement. However, if a simple mode of delivery is found for CRISPR/Cas9 for instance, such a method could be deployed widely if the cost is reasonable. The critical issue for gene therapy however is whether patients doing well on ART will accept gene modification with unknown risks for mutations, malignancies, and other potentially serious adverse effects.

### Intensification of antiretroviral therapy as a cure

The serendipitous report of the HIV-positive Mississippi baby who was put on ART 30 h after birth and was able to maintain viral suppression off treatment for 27.6 months raised the hopes that early ART could lead to a possible functional cure, especially if started early after the initial infection [30, 31]. A similar report was also observed in a French girl who has gone into remission for over 12 years after starting and interrupting ART at 3 months and 6.5 years old respectively [177]. Recently, a report from South Africa indicates that a child who started treatment at 2 months and discontinued it after 10 months has remained in remission for 9.5 years [178]. Using an analytic treatment interruption (ATI) method, remission was also observed in some children in South Africa who started ART as early as 14 days after birth [181]. Furthermore, in adults, it has been shown that initiating ART earlier results in HIV remission and smaller reservoir size [32, 179–184].

Bearing in mind that the norm is viral rebound within weeks of interrupting ART in the majority of HIV-infected persons, these findings highlight the potential benefits of early ART. Nonetheless, very early ART on its own is not likely to achieve sustained virologic remission in the majority of HIV-infected persons. A combination of early ART with other curative strategies such as broadly neutralizing antibodies, and therapeutic vaccines is likely to be the ultimate approach. Initiating early ART should be based on improving clinical outcomes rather than achieving remission [57, 185].

The main problem with this approach is that most patients in Africa and elsewhere live with HIV for years before they recognize they have the infection, by which time the reservoir is well established and ART alone will not result in long-term remission. Moreso, access to ART and funding still remains a challenge and thus its sustainability on the African continent is questionable.

### Immune-based interventions

HIV causes severe damage to the immune system of the host [186, 187], destroys CD4 T cells, and evades immune responses [188]. The aim here is to compensate for the loss of CD4 T cells [189], augment the anti-viral effects of CD8 + T cells (CTLs), and enhance neutralizing antibody-mediated killing of infected cells [190–192]. In this section, therapeutic vaccines, broadly neutralizing antibodies (bNab), and chimeric antigen receptors will be highlighted (Fig. 2). Therapeutic vaccines aim to increase the magnitude and function of anti-HIV immunity by facilitating long-term viral control without ART [193]. They are administered after a disease or infection has already occurred using the patient’s immune system to fight the infection [192]. In this case, they would produce HIV-specific immune responses to better control the virus when ART is interrupted [194] by eliciting anti-viral CD8 T cells (CTLs), and neutralizing antibodies [190–192, 195]. Therapeutic vaccines may also produce polyfunctional T cells which will release multiple cytokines and perform effector functions [196, 197].

Therapeutic vaccines are used to augment CTLs to increase their cytotoxicity capacity [198, 199]. CTLs are a major component of the host response to HIV [200–203] and are usually exhausted due to their persistent exposure to HIV which impairs their killing ability [204, 205]. Even during HIV control using ART, there is reduced virus-specific CD8 T cell responses [206, 207], implying that to control HIV, T cells need to be augmented. So far, therapeutic vaccine trials have failed to achieve functional HIV cure and sustained viral control after ART was stopped [208–211]

Research has also shown that broadly neutralizing antibodies (bNAbs) can control HIV replication [212–217]. All bnAbs target the HIV-1 Envelope (Env) glycoprotein 120 (gp120) and gp41 [218]. The first-generation bNabs (b12, 447-52D, 2G12, 17b, 2F5, 4E10 and Z13) [219–221] generated little clinical effect on HIV [222]. Next, antibodies targeting the CD4 binding site (VRC01, 3BNC117, VRC01-LS, and VRC07-523LS), the glycan-rich V3 loop (10–1074 and PGT121), the V2-glycan site (PGDM1400, CAP256-VRC26.25) and MPER epitope (10E8) were identified [218, 223, 224]. All these bNAbs have shown different levels of protection against Simian Immunodeficiency Virus (SHIV) [225–228]. Research has shown that combining two or more bNAbs is effective in enhancing a broad viral coverage [229–233]. bnAbs against HIV have shown significant promise for their potential use in the control of HIV, [218, 223], however, one setback is to identify combinations of bnAbs that will increase the breadth to cover circulating variants [218, 234]. In terms of HIV cure or remission, bNAbs could be given to patients who are virologically suppressed on ART to keep the virus undetected after withdrawal of ART. Issues of broad coverage against HIV variants and how long these antibodies last in vivo will be critical for success. They could also be deployed as part of a therapeutic vaccine strategy as discussed above.

Chimeric antigen receptors (CAR), following their breakthrough in the treatment of cancers [235–237], are currently being employed to enhance recognition and the killing of cells infected with HIV [69, 238]. They are produced by first removing patient T cells and inserting into them a CAR against a specific antigen [239] and then reinfusing the CAR-T cells into the patient [240], thus giving T cells a new ability to target a specific protein. This technology has seen the production of four generations so far [241–245]. The CAR T cell therapy can be used in conjunction with LRA to enhance the killing of reactivated cells, thus reducing the size of the reservoir [69].

Even though CAR-T cell therapy has proven to be beneficial, it faces some challenges. The cost of treating a single patient as well as the technical expertise in designing the therapy limits its use not only in Africa but even in the developed world [246].

Another important limitation is the lack of universality since they need to be produced from autologous T cells. More so, the long process in the design and treatment of patients makes it difficult to implement in developing countries [247]

These limitations notwithstanding, researchers are working to produce universal CAR T cells that could be taken off the shelf and given to any patient. It is expected that over time, the technology will mature, become easier to manage and less expensive.

### Which HIV cure strategies are appropriate for Africa?

It is worth mentioning that, none of the HIV cure strategies discussed here has been tried on the African soil. Only a few targeting therapeutic vaccines and HIV treatment have been tried in African as shown in Table 2.

**Table 2:** Some ongoing and completed clinical trials of HIV cure in Africa

Trial	Trial registry identifiers	Phase	Estimated end	Reference
*Ongoing clinical trials of HIV cure in Africa*				
VRC07-523LS, CAP256V2LS, vesatolimod	NCT05281510	Phase IIa	Feb-2024	[107]
IMPAACT P1115 v2.0: very early intensive treatment of HIV-infected infants to achieve HIV remission (ART + /– VRC01)	NCT02140255	Phase I/II	Dec-2031	[108]
*Completed clinical trials of HIV cure in Africa*				
VRC01 (broadly neutralizing antibody) in infants	NCT03208231	Phase I/II	Feb-2021	[107]
VRC01LS + 10–1074 (broadly neutralizing antibodies) in early-treated children	NCT03707977	Phase I/II	Oct-2021	[109, 110]
AFO-18 (peptide-based vaccine)	NCT01141205	Phase I	Jun-2012	[111]
VRC01 in acute HIV infection	NCT02591420	Phase I	Mar-2021	[107]

As cure strategies are developed, several factors inherent to the strategies themselves will determine how appropriate they are for Africa. These factors include the type of cure offered (functional versus complete), ease of deployment, perceived risks, and cost.

First, the type of cure offered, whether complete or functional could be important in determining patient acceptance. Ideally, a complete cure for HIV is desirable as it gives peace of mind to patients that the virus is gone. However, some form of functional cure is likely to be more feasible in the near future. How will PLWH in Africa perceive sustained remission or functional cure? Such a cure will mean patients need to have periodic follow-up. Moreover, will PLWH in Africa accept that they are ‘cured’ but there is still virus in the body as approaches like block and lock are likely to provide? Will they see sustained remission as a major improvement over daily ART, and or will anxieties about potential viral rebounds keep patients away from such remedies? These are important questions that need to be answered now before these remedies become realities. Knowing patient and caregiver perspectives on these issues could inform the design of clinical trials for remission strategies and ultimately the kind of cures made available to patients. Second, a cure that is easy to deploy is more likely to gain ground in Africa. Gene therapy, especially the type that requires pheresis of white cells from patients, modification and reintroduction will be expensive and logistically difficult to implement in Africa. When it comes to gene therapy, efforts should concentrate on developing delivery vehicles for methods like CRISPR/Cas9 for easy administration. Methods like shock and kill and block and lock or even therapeutic vaccines that require adding another agent to ART will be easier to deploy. Even for these, the route of administration will be important. Treatments that are given orally or simple intramuscular injections will be easier than methods that require admission and intravenous infusions at a medical center. Therefore, all these factors should be taken into consideration as scientists develop these cure strategies. Third, the perceived risks and benefits of the cure method offered in Africa will be critical in determining uptake or even participation in clinical trials. How will methods such as CRISPR/Cas9-mediated gene therapy that modify part of the patient’s genome be viewed in Africa? With the current COVID-19 pandemic, there is a lot of misinformation and resistance to mRNA vaccines in Africa even though they do not modify the genome. This will call for close collaboration and education for both providers and patients about trial methods prior to their availability in clinical trials. Such education and interactions may even help modify some of the methods that are eventually brought to patients. Finally, the cost of the intervention will also be crucial in ensuring wide deployment in Africa. In Africa, HIV treatment is heavily dependent on donor support with the constant threat that this support could be cut off. The ideal HIV cure for Africa should therefore be affordable, easy to administer, have short treatment time and as be low risk as possible. These factors should be at the forefront as scientists develop HIV cures. African scientists should be involved in the development and clinical trials of these strategies to engender confidence among patients. Of all the cure strategies discussed, the shock and kill block and lock, therapeutic vaccines, and perhaps CRISPR/Cas9-based treatment with easy delivery systems will be most appropriate for the African continent.

## Data Availability

Not applicable.
